# Oligosarcoma uniformly exhibits a HOX gene family hypermethylation signature

**DOI:** 10.1007/s00401-025-02905-3

**Published:** 2025-07-02

**Authors:** Vinny Ha, Nicholas Nuechterlein, Mia Rivers, Drew Pratt, Patrick J. Cimino

**Affiliations:** 1 Neuropathology Unit, Surgical Neurology Branch, National Institute of Neurological Disorders and Stroke, National Institutes of Health, 10 Center Drive, Building 10/3D17, Bethesda, MD 20892 USA; 2 Laboratory of Pathology, National Cancer Institute, National Institutes of Health, Bethesda, MD USA

Recently, several independent studies have shown that a subset of aggressive IDH-mutant adult-type diffuse gliomas display aberrant DNA methylation patterns across developmentally regulated HOX gene family members [3, 6–8]. Our group has previously shown that a large subset of oligodendroglioma, IDH-mutant and 1p/19q-codeleted are characterized by a pan-HOX gene body DNA hypermethylation signature [8]. Furthermore, HOX gene body DNA hypermethylation corresponds to increased gene expression in oligodendroglioma [8]. Oligosarcoma is a recently described aggressive IDH-mutant glioma subtype with its own distinct histological and DNA methylation features whose HOX gene methylation patterns have yet to be investigated [2, 5, 11]. Here we analyzed 52 high-confidence methylation‑defined oligosarcomas (DKFZ CNS v12b6 family class score > 0.89 and subclass score > 0.80; 16 profiled at the NIH aggregated with 36 publicly available samples from GSE190362, GSE237103, GSE248471, GSE260850, and TCGA) alongside 284 oligodendrogliomas from cohorts published by Capper et al. and TCGA [1]. DNA methylation IDAT files for these samples were processed in parallel and Uniform Manifold Approximation and Projection (UMAP) dimensionality reduction was applied to the beta values of the 317 methylation probes associated with the gene body regions of the 39 HOX gene family members as previously described [8]. These analyses reproduced our previously reported oligodendroglioma HOX-High and HOX-Low methylation groups, and UMAP projections showed that oligosarcoma samples are placed near HOX‑high oligodendroglioma samples, with considerable overlap between groups (Fig. 1a, b) [10]. Notably, pan-HOX gene body methylation was significantly higher in the oligosarcoma group compared to the HOX-low oligodendroglioma group (*p* < 0.0001, Mann–Whitney *U* test) (Fig. 1c). Differential methylation probe analysis between our oligosarcomas and oligodendrogliomas showed that *HOXB1* and *HOXB3* were the most significantly differentially methylated (Fig. 1d).

**Fig. 1:**
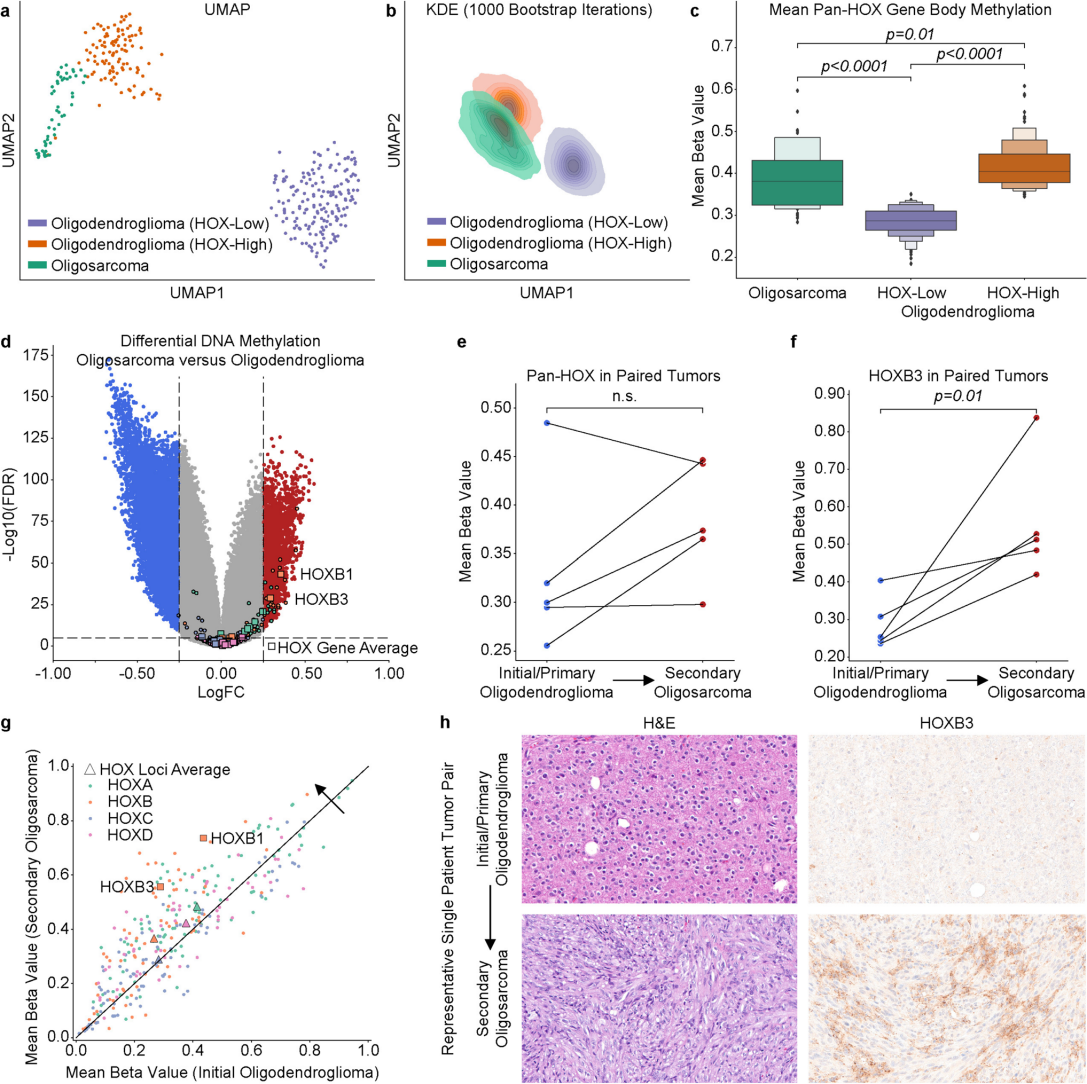
Pan-HOX gene body hypermethylation signature in oligosarcoma. **a** UMAP projection of pan-HOX gene body CpG sites for oligodendrogliomas (*n* = 284) and oligosarcomas (*n* = 52) shows oligosarcomas cluster singularly near the HOX-High oligodendroglioma group. **b** Kernel Density Estimation over 1000 bootstrapped UMAP projections confirms stability of HOX-based oligodendroglioma and oligosarcoma grouping. **c** Pan-HOX average gene body methylation is generally high in oligosarcoma and is more similar to the HOX-High than HOX-Low oligodendroglioma group. **d** Volcano plot of differentially methylated genes between oligosarcoma and oligodendroglioma. **e** Pan-HOX gene body methylation compared across paired tumor samples. **f** HOXB3 gene body methylation as an example compared across paired tumor samples. **g** Gene body relative hypermethylation in oligosarcoma occurs across all four of the HOX genomic clusters (A–D). **h** Representative histology and HOXB3 immunohistochemistry from a paired initial oligodendroglioma with its secondary oligosarcoma

We next analyzed five primary oligodendroglioma tumors known to have recurred as oligosarcoma. Four of the five initial oligodendrogliomas were characterized as HOX-low tumors, of which three had increased HOX gene body methylation at recurrence (Fig. 1e). HOXB3 was significantly hypermethylated in secondary oligosarcoma compared to their primary oligodendroglioma (Fig. 1f). Although underpowered, a granular analysis at the 328 individual HOX gene body CpG sites showed that most probes associated with each HOX genomic locus had higher methylation levels in recurrent oligosarcomas compared to their matched initial oligodendroglioma tumor (Fig. 1g). Of the four HOX loci, genes in the HOXB locus (located on chromosome 17) had the highest level of gene body DNA methylation in the recurrent oligosarcomas (Fig. 1g). Ancillary HOXB3 immunohistochemical staining (Invitrogen, #16610345, rabbit polyclonal, 1:100, ER2) performed on a Leica autostainer as previously described [9] highlights increased HOX activation in a paired secondary oligosarcoma (Fig. 1h).

In summary, oligosarcomas demonstrate a HOX gene body hypermethylation signature, similar to the aggressive HOX-High subset of oligodendrogliomas previously reported [8]. Furthermore, we demonstrate preliminary evidence that HOX gene body methylation may increase during progression from oligodendroglioma to oligosarcoma. HOX genes, such as HOXB3, are known to play roles in cancer promotion, proliferation, and invasion, supporting the biological role of an aggressive phenotype for oligosarcoma [4]. Overall, the uniformity of oligosarcoma HOX gene body methylation profile and its similarity to an aggressive form of oligodendroglioma further indicates that these tumors are generally among the most aggressive types of IDH-mutant gliomas [3, 6–8].

## Data Availability

Data will be made available upon request.
